# Preoperative resting‐state microstate as a marker for chronic pain after breast cancer surgery

**DOI:** 10.1002/brb3.3196

**Published:** 2023-07-26

**Authors:** Yaru Li, Lu Wang, Qiaoyu Han, Qi Han, Luyang Jiang, Yaqing Wu, Yi Feng

**Affiliations:** ^1^ Department of Anesthesiology Peking University People's Hospital Beijing China; ^2^ Key Laboratory of Carcinogenesis and Translational Research Ministry of Education Beijing China; ^3^ Department of Anesthesiology Peking University Cancer Hospital & Institute Beijing China; ^4^ Department of Pain Medicine Peking University People's Hospital Beijing China; ^5^ Key Laboratory for Neuroscience Ministry of Education of China and National Health Commission Beijing China

**Keywords:** electroencephalography (EEG), microstate, postoperative chronic pain

## Abstract

**Introduction:**

Chronic postoperative pain poses challenges, emphasizing the importance of accurately predicting pain in advance. Generally, pain perception is associated with the temporal dynamics of the brain, which can be represented by microstates. Specifically, microstates are transient and patterned brain topographies formed by temporally overlapping and spatially synchronized oscillatory activities. Consequently, by characterizing brain activity, microstates offer valuable insights into pain perception.

**Methods:**

In this prospective study, 66 female patients undergoing breast cancer surgery were included. Their preoperative resting‐state electroencephalography (EEG) was recorded. Preoperative resting‐state EEG was recorded and four specific brain microstates (labeled as A, B, C, and D) were extracted. Temporal characteristics were then analyzed from these microstates. Patients were classified into two groups based on their Numerical Rating Scale (NRS) scores at three months postoperatively. Those with NRS scores ranging from 4 to 10 were classified as the high pain group, while patients with NRS ranging from 0 to 3 were classified as the lowpain group. Statistical analyses were performed to compare the microstate characteristics between these two groups.

**Results:**

Twenty‐one patients (32%) were classified as the high pain group and forty‐five (68%) as the low‐pain group. The occurrence and coverage of microstate C were significantly higher in the high pain group. Additionally, there were significant differences in the microstates transitions between the two groups. Furthermore, the study revealed a positive correlation between the coverage of microstate C and the NRS.

**Conclusions:**

Preoperative resting‐state microstate features have shown correlations with postoperative pain. This study presents a novel and advanced perspective on the potential of microstates as a marker for postoperative pain.

## Graphical abstract

The study collected resting‐state EEG data from 66 patients with breast cancer prior to surgery, and the results showed significant differences in the occurrence, coverage, and transition of microstate C in patients who developed severe chronic pain after surgery. This study provides a new approach for using resting‐state EEG to predict postoperative pain in breast cancer patients.

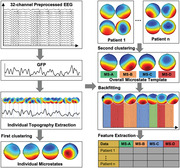

## 1 INTRODUCTION

According to the International Association for the Study of Pain (IASP), postoperative chronic pain is defined as pain that occurs after surgery, which is related to the surgical site, and persists for at least three months or more (Schug et al., 2019). Postoperative pain is a widespread problem, with an incidence rate of 20–65% in breast cancer surgery (Schreiber et al., 2021). Chronic pain following breast cancer surgery can cause sensitive and sensory disorders in various regions, including the chest wall, axilla, and breast. This could significantly affect the life quality of patients and thus need treatment. Treatment for chronic pain might be a long and challenging process, highlighting the importance of preventive measures for postoperative pain (Okamoto et al., 2018; Sessler et al., 2019). Risk factors for postoperative pain typically encompass a combination of subjective factors such as anxiety, depression, and pain expectations (Schreiber et al., 2021). Moreover, the individual roles of these factors in postoperative pain remain unknown, resulting in the prediction of postoperative pain a significant challenge. Therefore, there is an urgent need in clinical practice for an objective and convenient method to estimate postoperative pain (Rehberg et al., 2017).

Previous research has demonstrated that the dynamics of electroencephalogram (EEG) serve as a crucial marker for pain (Mouraux & Iannetti, 2018; Peirs & Seal, 2016). However, most studies typically focus on the static measures, such as gamma band of brain oscillations, while neglecting the dynamic nature of the brain (Zis et al., 2022). There is an increasing recognition that the dynamic temporal information of brain activity may reflect individual pain sensitivity and contribute to the interindividual differences in pain perception (Hu & Iannetti, 2019; Ta Dinh et al., 2019). To comprehensively investigate pain‐related information at the level of brain networks, microstate analysis has emerged as one of the most advanced approaches (Mishra et al., 2020). Microstates are transient and patterned brain topographies characterized by stable scalp potential fields for approximately 80–120 ms before transitioning rapidly to a different topography. They involve temporally overlapping and spatially synchronized oscillatory activities (Khanna et al., 2015; Michel & Koenig, 2018). Consequently, EEG microstates are regarded as a valuable tool for assessing brain function by providing highly precise temporal information at a sub‐second timescale. Four classic microstate topographies are commonly classified as microstate A, B, C, and D. Microstate A is related to the auditory network, while microstate B is related to the visual network. Microstate C is associated with the salience network, which includes the activation of the anterior cingulate cortex (ACC) and insula. Additionally, microstate D is believed to be involved in the attention network (Michel & Koenig, 2018). Studies have proposed several abnormal manifestations of microstates in chronic pain patients (Gonzalez‐Villar et al., 2020). May et al. (2021) found that compared to healthy volunteers, patients with chronic pain exhibit lower time coverage and occurrence of microstate D. Zhou et al. (2023) conducted a study that combing the analysis of resting‐state microstates and functional magnetic resonance imaging (fMRI) to investigate brain network changes of migraine patients. The study revealed significant alterations in the brain network of individuals with migraines. Another study revealed that microstate C is relatively less active in fibromyalgia patients compared to healthy individuals (Gonzalez‐Villar et al., 2020). Despite variations in research findings, there is collective evidence indicating a reorganization of brain network connections in individuals with chronic pain. Although many studies have focused on occurred pain, there is limited research dedicated to exploring the latent information within pre‐pain microstates. Furthermore, there is currently no consensus on whether resting‐state microstates prior to surgery can accurately assess postoperative pain.

Therefore, this study aimed to investigate whether resting‐state microstate prior to surgery could serve as a marker for evaluating chronic pain following breast cancer surgery.

## 2 METHODS

### 2.1 Participant

The study enrolled female patients who were scheduled for breast‐conserving surgery or mastectomy under general anesthesia. Inclusion criteria were ages between 18 and 70 years old, with a physiological status categorized according to the American Society of Anesthesiologists (ASA) classification system as ASA stage I and II, body mass index (BMI) 18–30 kg/cm^2^, right‐handed. Exclusion criteria included distant metastasis of breast cancer, history of psychiatric or neurological disorders or related medication use, bilateral surgery, history of other chronic or acute pain conditions, and inability to communicate. All patients were followed up for up to 3 months after discharge. The study was conducted at Peking University People's Hospital and it was approved by the Institutional Review Board of the institution (Ethics Committee of Peking University People's Hospital, approval number 2022PHB022‐001). The study was registered at the Chinese Clinical Trial Registry (ChiCTR2200065214). Written informed consent was obtained from all participants.

### 2.2 Data collection

Demographic information was collected, including sex, age, BMI, and education. Surgery‐related data were collected, including ASA stage, the history of preoperative chemotherapy, surgery type, axillary lymph node dissection (ALND), and postoperative chemotherapy. Notably, all patients underwent a preoperative evaluation for depression utilizing the Beck Depression Inventory II (BDI II). The observation period was set at three months postoperatively. Chronic postoperative pain was defined as pain persisting for a duration of 3 months following surgery in the operated breast, encompassing the shoulder, chest wall, axilla, or upper arm. Pain assessment was conducted using the standard 11‐point Numerical Rating Scale (NRS, 0 = “no pain” and 10 = “the worst possible pain”). Patients were asked to rate their average pain and complete the Brief Pain Inventory questionnaire (BPI) over the telephone 3 months after surgery. In clinical practice, pain with an NRS score greater than 3 is recommended for treatment (Gerbershagen et al., 2011). Therefore, in the study, patients with an NRS score ranging from 4 to 10 were allocated to the high pain group (HP group), while those with an NRS score of 0 to 3 were categorized as the low‐pain group (LP group). This is consistent with previous research classification (Yao et al., 2022). All pain assessments were conducted by a professional independent follow‐up worker.

### 2.3 Procedure and EEG recording

At 9 AM to 10 AM on the day before the surgery, patients were placed in a quiet and comfortable environment and briefly introduced to the EEG recording. EEG data were recorded using a 32‐channel Neuroscan System consisting of all 10−20 system electrodes (Compumedics Neuroscan, Inc.). The sampling rate of the NuAmps amplifier (model 7181) was 1000 Hz, and the data were bandpass filtered between 0.01 to 100 Hz. Impedances were kept below 10 kΩ. Patients were instructed to stay awake and relax while their EEG was recorded for 5 min during both eyes‐closed and eyes‐open conditions.

### 2.4 EEG preprocessing

The EEG data were preprocessed offline using the EEGLAB toolbox v14.1.1 (Delorme & Makeig, 2004) in Matlab R2022b (The MathWorks Inc, Natick, MA, USA). The EEG data were digitally bandpass filtered between 0.1 and 80 Hz, and the 48–52 Hz power line noise was removed. The data were then resampled to 500 Hz. The two mastoid electrodes (M1, M2) were averaged to serve as a reference, and the entire recording was preliminarily screened by professionals, with heavily contaminated segments marked as “bad segments.” If a channel was found to be damaged, bad channel interpolation was performed. Subjects were excluded if more than three channels in their EEG data were damaged. Additionally, independent component analysis was conducted to eliminate residual noise from eye blinks, muscle activity, and electrode artifacts (Pedroni et al., 2019).

### 2.5 Microstate analysis

**FIGURE 1:**
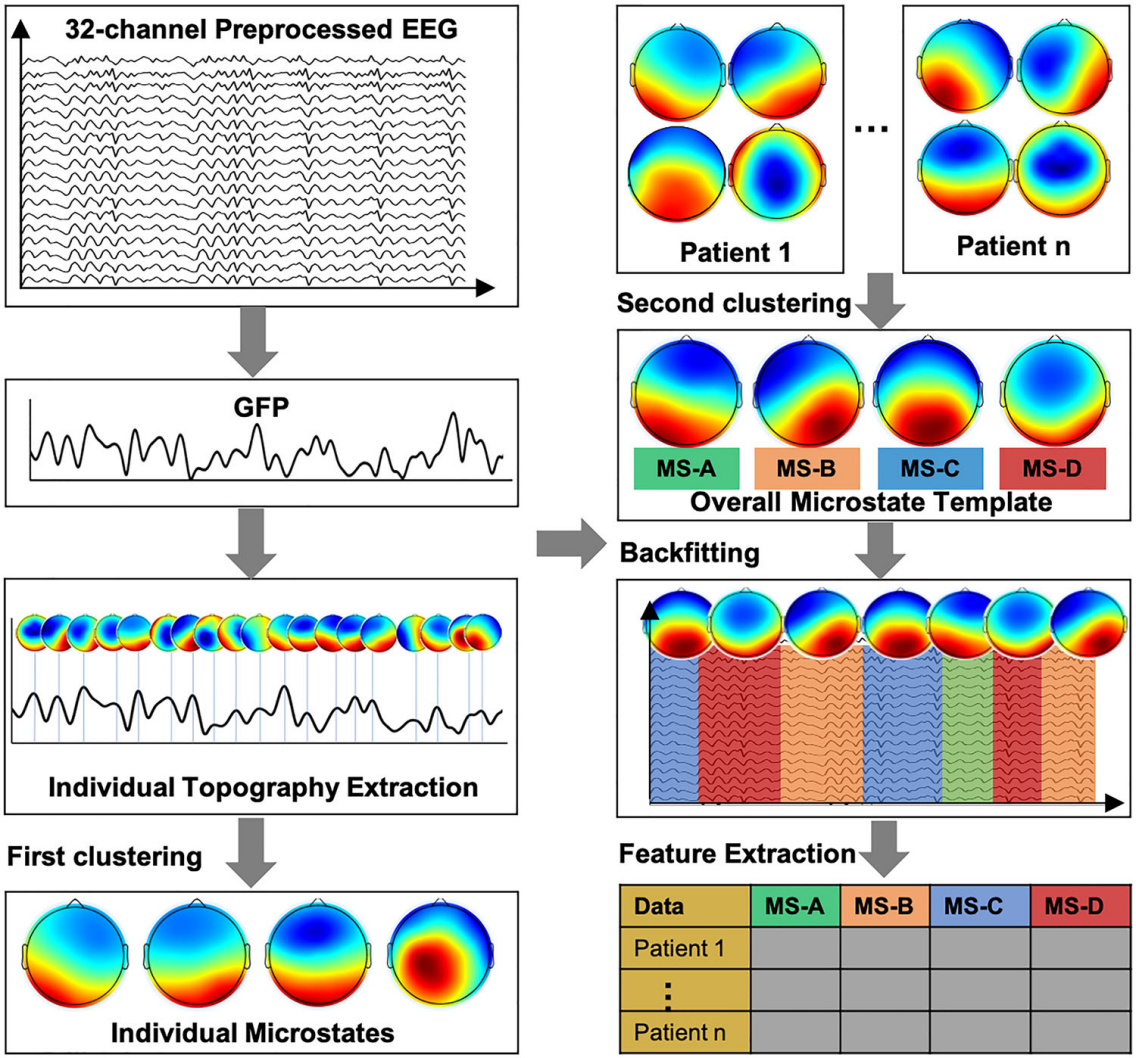
Microstate analysis process flowchart. For each preprocessed EEG, the Global Field Power (GFP) is calculated and topographies are extracted at the peaks of GFP. Then, the first *k*‐means clustering at the individual level is undergone by the generated topographies, resulting in four individual level microstates. Subsequently, a second clustering is performed across individuals to generate the overall microstate templates labeled as MS‐A to MS‐D. These templates are then fitted back to the EEG data, enabling the extraction of temporal characteristics of microstates.

Microstate analysis employs the use of MATLAB, as well as its associated plugins including EEGLAB, FieldTrip (Donders Institute for Brain, Cognition and Behaviour at Radboud University, Nijmegen), and relevant scripts (Oostenveld et al., 2011). The entire process of microstate analysis adheres to the recommended standards and protocols established in the research field. http://www.thomaskoenig.ch/index.php/work/ragu. In general, microstate analysis is conducted using a two‐step *k*‐means clustering method, where the first clustering is performed at the individual level to segment the EEG data and identify the most representative template maps. Subsequently, a second clustering is performed across individuals to generate the overall microstate templates. These templates are then fitted back to the EEG data, enabling the extraction of microstate sequences. As shown in Figure 1, the schematic diagram illustrates the process of microstate analysis. First, for each patient, the global field power time series (GFP) is computed as the spatial standard deviation of the EEG topography. At the time points corresponding to GFP peaks, all EEG topographies are extracted, yielding a variable number of individual‐level EEG topographies. To mitigate noise, peaks surpassing a threshold of two times the standard deviation of all GFP peaks are excluded. Subsequently, microstates are identified utilizing a modified *k*‐means algorithm, disregarding polarity. For each patient, four random microstates were chosen as the initial cluster centers. The difference between each original topography and the initial cluster centers is calculated, assigning them to the most similar cluster center. The mean of all data within the generated set is computed and used as the new cluster center. Clusters are ultimately selected and extracted as representative of the individuals based on their high global explained variance (GEV) and low cross‐validation (CV) criterion values. These steps are iterated until the cluster centers converge or reach the predefined iteration limit. The initial value of k is set to four, and the iteration is performed 100 times (Khanna et al., 2015; Hu et al., 2023).

During the second step, a second *k*‐means clustering was performed by clustering the merged individual topographies obtained in the previous step. This resulted in the generation of four microstate templates (labeled as MS‐A, B, C, and MS‐D) across the entire patient population, which were consistent with previous classical studies (Musaeus et al., 2020). Following the acquisition of the overall microstate templates, they were fitted back into the original EEG signals of each individual, thereby establishing a continuous sequence of microstates at the temporal scale. The temporal characteristics of each microstate were quantified using four parameters computed from the microstate time series: duration, occurrence frequency, coverage, and transition probabilities.

#### 2.5.1 Duration

The duration refers to the length of time that an individual microstate persists.

#### 2.5.2 Occurrence

Occurrence represents the frequency of a microstate appearing in the entire sequence.

#### 2.5.3 Coverage

Coverage indicates the proportion of time occupied by a microstate in the sequence.

#### 2.5.4 Transition

Transition denotes the change from one microstate to another within the sequence (Chu et al., 2020).

### 2.6 Statistics

The statistical analysis was performed using SPSS 22.0 software (SPSS Statistics, IBM) and MATLAB (The MathWorks Inc). Continuous variables were presented as mean ± standard deviation, while categorical variables were presented as percentages. The Shapiro–Wilk test was conducted to evaluate the normality of continuous variables. For normally distributed variables, independent sample *t*‐tests were employed to compare differences between two groups. For variables that were not normally distributed, the Mann–Whitney *U* test was used to compare differences between the two groups. Chi‐square test was used for comparison of categorical variables between groups. A Generalized Linear Model (GLM) was used to control for potential influences of clinical characteristics on the results of group comparisons and correlation analysis. The GLM included age, surgery type, BDI‐II, and ALND as covariates. Analysis of variance analysis (ANOVA) was then conducted to test for differences in three parameters (duration, occurrence, coverage), with microstate class as the within‐group variable and whether or not chronic pain occurred was used as a between‐group variable. In cases where main or interaction effects were significant, independent sample *t*‐tests were used to examine the parameters. The analysis of transition probabilities was also conducted using independent samples *t*‐tests. The relationship between the NRS and microstate parameters was assessed using Pearson correlation. Cohen's *d* was computed for independent samples *t*‐tests to estimate the effect sizes of significant results. For the comparison of microstate parameters between the two groups, the false discovery rate (FDR) was employed to correct for multiple comparisons on the original *p* values, and two‐tailed *p* values less than .05 were considered statistically significant, corrected *p* are reported.

## 3 RESULTS

### 3.1 Demographic and clinical characteristic

This study enrolled a total of 70 patients, of whom 2 cases were excluded due to poor EEG signal quality and 2 cases were lost to follow‐up, resulting in a total of 66 participants completing the study. Among the study cohort, 21 (32%) suffered from chronic postoperative pain with NRS score greater than 3 (HP group) and 45 (68%) were categorized as low‐pain patients with an NRS of 0–3 (LP group). Table 1 presents the basic demographic and clinical characteristics of all patients. There were no significant differences in age, BMI, ASA stage, education, BDI‐II, history of preoperative chemotherapy, postoperative chemotherapy between the two groups. However, there was a significant difference between the two groups in terms of the type of surgery. Mastectomy was associated with higher postoperative pain compared to breast‐conserving surgery (*p* = .041), and the NRS of patients was significantly higher for those who had underwent undergone ALND (*p* = .011).

**TABLE 1:** Demographic data and clinical characteristics.

	HP (mean ± SD)	LP (mean ± SD)	*p*
Number	21	45	
Age (years)	52.7 ± 9.9	54.5 ± 8.4	.458
BMI (mean)	24.7 ± 3.8	24.2 ± 2.9	.554
Education (years)	10.9 ± 2.6	11.0 ± 2.8	.844
BDI‐II	5.3 ± 4.3	3.3 ± 2.3	.055
ASA I/II	6/15	16/29	.575
Preoperative Chemotherapy (Y/N)	9/12	13/32	.262
Surgery^a^			**.041**
Breast‐conserving surgery	10	33	
Mastectomy	11	12	
ALND (Y/N)^a^	14/7	15/30	**.011**
Postoperative Chemotherapy(Y/N)	11/10	18/27	.345

BMI: body mass index; ASA: American Society of Anesthesiologists; BDI‐II: Beck Depression Inventory II; HP: high pain group; LP: low‐pain group; ALND: axillary lymph node dissection.

^a^ *p* < .05.

### 3.2 Microstate analysis

**FIGURE 2:**
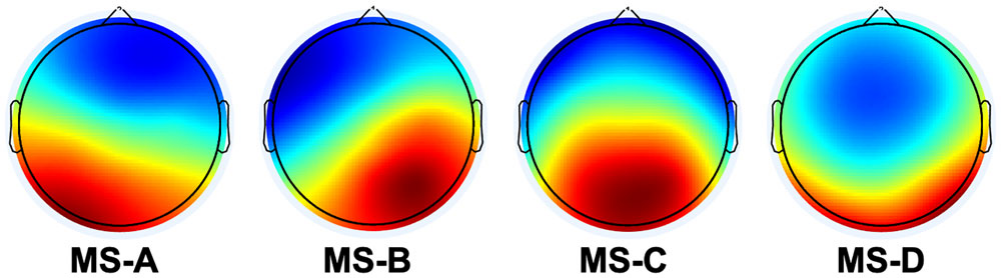
The overall microstate templates generated across all patients.

**FIGURE 3:**
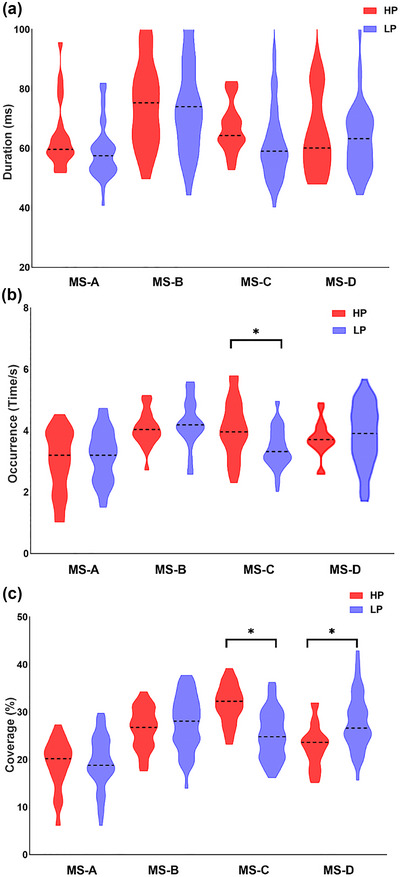
Comparison of the microstate temporal dynamics between two groups. (a) The mean duration of each microstate class in each of the two groups. No difference was found in the two groups. (b) The occurrence of each microstate class in each of the two groups. (c) The coverage of each microstate class in each of the two groups (after correcting for the confounding effects of age, surgery type, BDI‐II, and ALND by GLM). MS: microstate, HP: high pain group, LP: low‐pain group. *p < .05.

**FIGURE 4:**
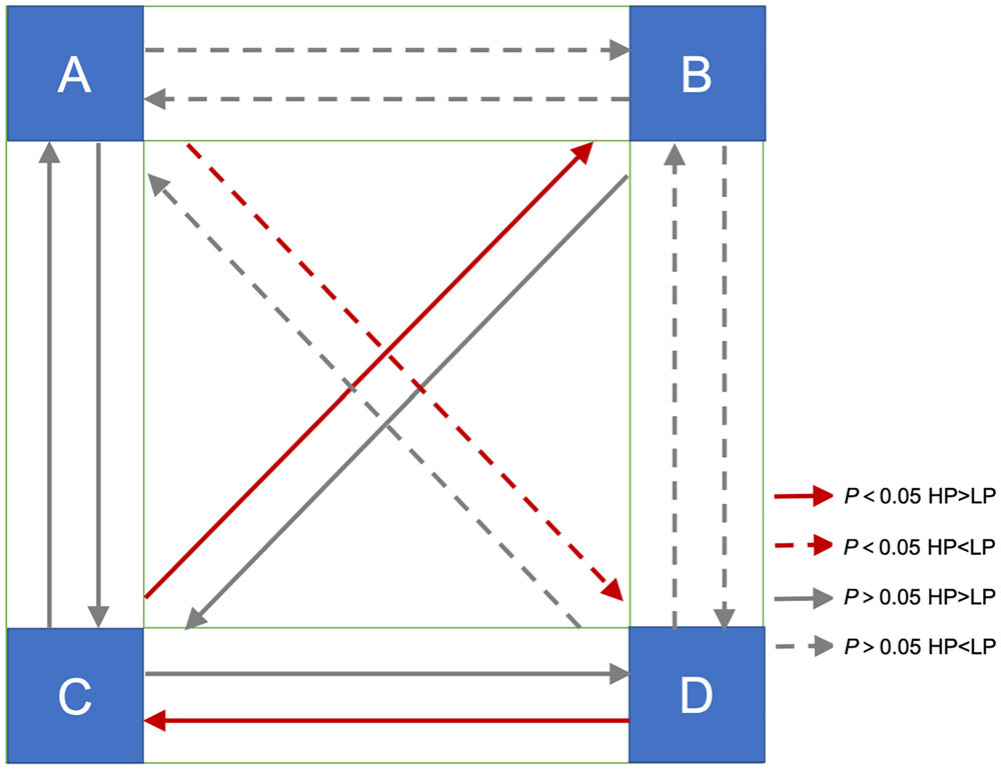
Comparison of the transition probability between four microstates. The solid arrows indicate a higher transition probability in the HP group compared to the LP group. The dashed arrows indicate a lower transition probability in the HP group compared to the LP group. The red color represents statistically significant differences (*p* < .05).

With reference to the classical microstate map described in previous study (Michel & Koenig, 2018), the microstates were categorized as MS‐A, B, C, and MS‐D (Figure 2). The mean GEV of the four classes was 0.814. The results indicated that there was no significant difference in duration among the four microstates in either group (Figure 3a). The independent sample *t*‐tests revealed that the occurrence of MS‐C was significantly higher in the HP group than in the LP group (corrected *p* = .016) (Figure 3b). Using a similar strategy for coverage analysis, we observed that the coverage of MS‐C was significantly higher in the HP group than in the LP group (corrected *p* < .001). The coverage of MS‐D was significantly lower in the HP group than in the LP group (Figure 3c). A detailed summary of these results is provided in Table 2. Regarding transition probability, we observed more transitions from C to B, D to C, and less transitions from A to D in the HP group compared with the LP group (Table 3, Figure 4).

**TABLE 2:** Comparisons of temporal microstate measures between two groups.

	HP		LP			
	mean	SD	mean	SD	Corrected *p*	Cohen's *d*
**Duration (ms)**						
**MS‐A**	63.90	11.27	59.00	9.20	.153	/
**MS‐B**	76.71	17.84	78.48	20.43	.869	/
**MS‐C**	66.96	8.54	62.14	13.62	.258	/
**MS‐D**	66.77	17.36	66.01	17.51	.908	/
**Occurrence (time, s)**						
**MS‐A**	2.97	1.05	3.18	0.84	.520	
**MS‐B**	4.17	0.47	4.26	0.76	.480	
**MS‐C** ^a^	4.17	0.81	3.51	0.69	**.016**	**0.88**
**MS‐D**	3.56	0.69	3.87	0.99	.758	
**Coverage (%)**						
**MS‐A**	18.61	5.98	18.81	5.72	.908	
**MS‐B**	27.11	4.63	28.89	9.90	.448	
**MS‐C** ^a^	31.58	6.22	26.08	9.26	**<.001**	**0.70**
**MS‐D** ^a^	22.69	5.58	26.22	10.49	**.040**	**–0.42**

^a^ *p* < .05.

**TABLE 3:** Comparisons of transition probabilities between microstates in two groups.

	HP		LP			
	Mean	SD	Mean	SD	Corrected *p*	Cohen's *d*
**A to B**	7.35	2.59	8.09	2.56	.448	
**A to C**	6.59	2.62	6.42	1.84	.869	
**A to D** ^*^	5.75	1.64	7.10	2.15	**.040**	**–0.71**
**B to A**	7.40	2.76	8.08	2.74	.494	
**B to C**	11.45	3.14	9.91	3.94	.240	
**B to D**	9.06	2.38	11.66	4.73	.053	
**C to A**	8.31	3.84	6.26	1.93	.072	
**C to B** ^*^	11.17	2.87	8.89	2.69	**.016**	**0.82**
**C to D**	8.04	2.36	8.02	2.56	.980	
**D to A**	5.72	2.04	6.93	1.84	.053	
**D to B**	8.22	2.20	10.41	3.71	.053	
**D to C** ^*^	10.95	2.85	8.23	2.39	**<.001**	**1.03**

^*^ *p* < .05.

### 3.3 Correlation between microstate and clinical characteristics

**FIGURE 5:**
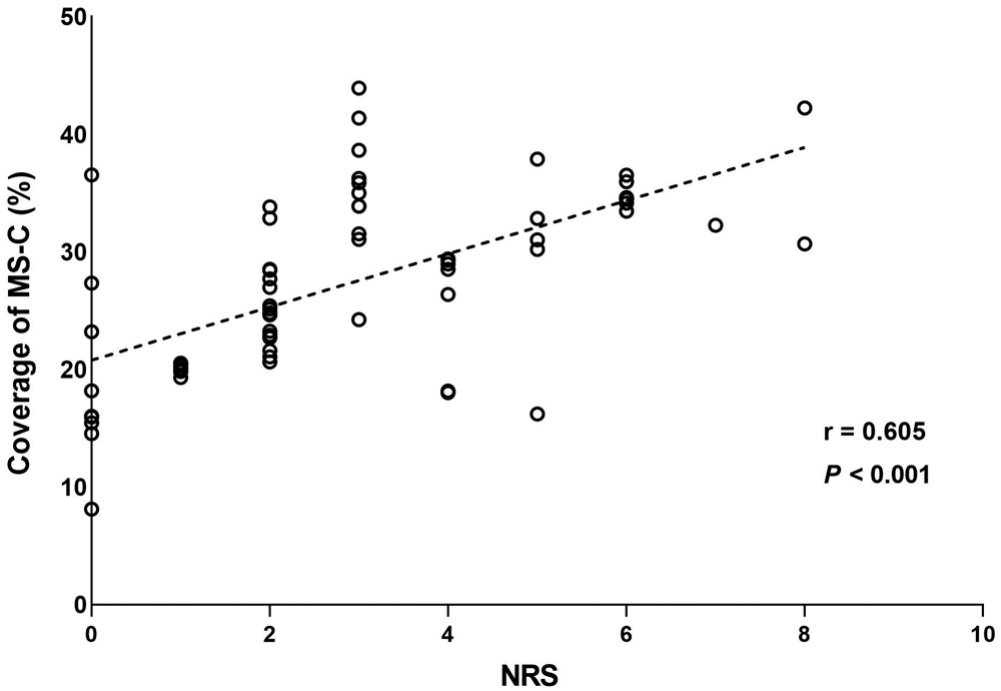
Correlations between microstate C and NRS. The coverage of microstate C is significantly correlated with NRS.

The Generalized Linear Model (GLM) showed no significant correlation between microstate parameters and clinical characteristics. Finally, Spearman correlation analysis indicated that the coverage of MS‐C had the strongest correlation with the NRS score (*r* = 0.605, *p* < .001) (Figure 5).

## 4 DISCUSSION

In this study, we performed an overall clustering of microstates in all patients and subsequently extracted temporal features of the microstates for between‐group statistical analysis. The results of our study unveiled that patients who are prone to postoperative pain exhibit different characteristics in MS‐C, including a higher occurrence and coverage in MS‐C, along with a greater frequency of transitions between MS‐C and MS‐D. Furthermore, we also observed a positive correlation between the coverage of MS‐C and NRS. The observed deviations in MS‐C among the HP group may indicate its potential involvement in pain perception.

Microstates are commonly referred to as the “atoms of thought” due to their reflection of distinct dynamic brain network activity. Due to their high reliability and stability, microstates account for over 90% of the EEG state (Lehmann et al., 1987; Mayaud et al., 2019). Morphologically, MS‐C characterized by topographical dominance of posterior activity, spanning from the frontal to occipital regions (Yuan et al., 2012). Typically, MS‐C manifests the activity of the salience network (Gan et al., 2023; Jiang et al., 2021; Li et al., 2022). The salience network can be regarded as a neural circuitry that includes the anterior insula (AI) and anterior cingulate cortex (ACC), anatomically (Menon, 2015; Seeley, 2019). Functionally, the salience network plays a fundamental role in detection and processing of salient stimuli originating from both internal and external. Additionally, it participates the pain connectome and undergoes further reorganization during chronic pain (Baumbach et al., 2022; Kucyi & Davis, 2015). Some fMRI studies (Cheng et al., 2017) have found that activity in the salience network is related to the cognitive processing of pain stimuli in a safe environment. Specifically, when participants directed their attention towards potential pain without actual painful stimuli, the salience network exhibited higher activity compared to individuals who did not focus on pain (Kim et al., 2021). The changes in the salience network activity are often reflected in the temporal characteristics of the MS‐C. Similarly, our research demonstrated that patients who experienced postoperative pain exhibited higher occurrence and coverage of MS‐C before the surgery. It can be inferred that differences in patients' cognition of pain result in variations in their attention towards pain. Clearly, those who pay more attention to pain are more likely to experience postoperative pain. These individuals have a more active salience network, which is reflected in more active MS‐C.

The current consensus is that pain competes for attention with other cognitive tasks. Research has found that patients with chronic pain often suffer from attention deficits, resulting in poor performance on tasks that demand a high level of attention (Bushnell et al., 2013; Kucyi & Davis, 2015). As mentioned previously, the attention network is generally considered to be associated with MS‐D from a microstate perspective. Recent research has discovered a significant correlation between the duration and coverage of MS‐D and pain intensity during sustained pain induced by capsaicin (Qiu et al., 2023). Another study (May et al., 2021) also found fewer occurrences of MS‐D in patients with chronic pain. These two studies indicated that after the occurrence of pain, patients might experience changes in the microstate dynamics (MS‐D). Unlike these studies that focus on occurred pain, our study mainly investigates the nonpainful population and has discovered similar findings. Our study found that the coverage of MS‐D was slightly lower in the HP group compared to the LP group. Another finding was a slightly more transitions between the MS‐C and MS‐D in the HP group, indicating an enhanced connectivity between the salience network and the attention network in this population. This abnormal transition between the two crucial resting‐state brain networks has also been observed in patients with complex regional pain syndrome, where an increased connectivity between the salience network and the attention network was found (Kim et al., 2018). In summary, we believe that the salience network is involved in pain cognition by responding to stimuli during nonpainful condition, while experiencing pain, the interaction between the salience network and the attention network becomes more prominent.

In addition to pain, previous study also found a potential relationship between microstates and other clinical characteristics. A recent study (Qin et al., 2022) indicated a significant discrepancy in MS‐B among undergraduates with high levels of depressive symptoms. The study also revealed a positive correlation between the MS‐B and Beck Depression Inventory II (BDI‐II) scores. However, our own research yielded no association between BDI‐II scores and microstate parameters, plausibly due to the fact that the average BDI‐II score among our patients was notably lower than 13, and only a single patient scored above this threshold (BDI‐II 14). Our research was obtained in painless and resting states, thus eliminating potential variability associated with task performance. Meanwhile it is commonly acknowledged that visual stimuli can cause interference when eyes are open (Zhou et al., 2020). Therefore, our analysis focuses on the EEG microstate during closed‐eye conditions. Studying eyes‐closed and resting‐state EEG allowed us to observe the characteristic brain dynamics associate with pain processing, which is crucial for evaluating postoperative pain.

There were several limitations in the current study. First, this study was conducted at a single center with a relatively small sample size, and the study population was limited to female participants, which may restrict the generalizability of the findings. Additionally, EEG contains rich information, and in this study, we only analyzed one dimension without incorporating other parameters. It is believed that by integrating EEG microstate with other neurophysiological metrics such as oscillations, coherence, and phase‐locking, we can gain a better understanding of how the brain processes information across various levels of neural networks. Moreover, the internal connectivity of the salience network is associated with higher pain sensitivity but we have only observed a phenomenon without conducting in depth analysis of the connectivity within the brain networks. Hence, despite the current findings that suggest a possible association between EEG microstate and pain sensitivity, the nature of such relationships is inherently intricate and may not conform to a linear pattern. There remain numerous brain mechanisms pertaining to pain sensitivity undiscovered. Further, more researches are needed to determine the universal applicability of pain‐related brain biomarkers and their effectiveness at the individual level (Tan & Kuner, 2021).

## 5 CONCLUSION

Our study unveils the presence of distinct temporal characteristics of microstates in individuals who are susceptible to postoperative pain, thus emphasizing the correlation between pain and the temporal dynamics of microstates. These findings provide evidence supporting the idea that EEG microstates act as a window into uncovering specific differences in pain‐related brain functional networks. In summary, this study presents a novel perspective on the potential application of preoperative resting‐state microstates as markers for postoperative pain following breast cancer surgery.

## AUTHOR CONTRIBUTIONS

YL contributed to conception and design of the study, organized the database, and wrote the first draft of the manuscript. YF contributed to conception and design of the study and manuscript revision. LJ completed the manuscript review and editing. QH and LW collected the data and performed the statistical analysis. YW conducted a follow‐up on postoperative pain. All authors contributed to manuscript revision, read, and approved the submitted version.

## FUNDING

This study was supported by National Key Research and Development Program of China, grant number being 2018YFC2001905.

## CONFLICT OF INTEREST STATEMENT

The authors declare that the research was conducted in the absence of any commercial or financial relationships that could be construed as a potential conflict of interest.

## DATA STATEMENT STATEMENT

The data that support the findings of this study are available on request from the corresponding author.

### PEER REVIEW

The peer review history for this article is available at https://publons.com/publon/10.1002/brb3.3196.
